# Editorial: Advancements in nutritional management for patients with renal failure

**DOI:** 10.3389/fmed.2026.1806351

**Published:** 2026-03-02

**Authors:** Shuping Song, Feng Guo, Zhongheng Zhang

**Affiliations:** 1 Intensive Care Unit, Sir Run Run Shaw Hospital, School of Medicine, Zhejiang University, Hangzhou, China; 2 Department of Emergency Medicine, Sir Run Run Shaw Hospital, Zhejiang University School of Medicine, Hangzhou, China; 3 Key Laboratory of Precision Medicine in Diagnosis and Monitoring Research of Zhejiang Province, Sir Run Run Shaw Hospital, Zhejiang University School of Medicine, Hangzhou, China; 4 School of Medicine, Shaoxing University, Shaoxing, China; 5 Longquan Industrial Innovation Research Institute, Lishui, China

**Keywords:** Appendicular Skeletal Muscle Mass (ASM), chronic kidney disease (CKD), Dietary Inflammatory Index (DII), nutritional management, Retinol-Binding Protein 4 (RBP4)

Chronic kidney disease (CKD) has been recognized as a leading public health problem worldwide. The global estimated prevalence of CKD is 13.4% (11.7–15.1%), and patients with end-stage kidney disease (ESKD) needing renal replacement therapy is estimated between 4.902 and 7.083 million (1). As nutritional management of patients with CKD is thought to control uremic symptoms and provide beneficial effects on the progression of kidney dysfunction, the diet of patients with CKD should be an important consideration in their care (2). Currently, the nutritional management of CKD has evolved from traditional protein and salt restriction toward multi-dimensional and precise interventions. Based on recent clinical studies, this editorial proposes an assessment framework integrating novel biomarkers such as the Dietary Inflammatory Index (DII, Li, Xu et al.), Retinol-Binding Protein 4 (RBP4, Lee et al.), and Appendicular Skeletal Muscle Mass (ASM, Romejko et al.). It emphasizes the establishment of an individualized nutritional management pathway—“assessment-intervention-reassessment”—through dynamic monitoring of inflammatory status, nutritional metabolism, and body composition, aiming to improve patient prognosis.

## 1 Breaking through the limitations of traditional nutritional assessment to establish a multi-dimensional biomarker system

The visceral proteins albumin and prealbumin must be correctly recognized as inflammatory markers associated with “nutrition risk” in nutrition assessment. Serum albumin and pre-albumin levels do not serve as proxy measures of total body protein or total muscle mass and are not useful monitoring parameters to guide nutrition support therapy. To identify these as markers of malnutrition is an oversimplification that should be avoided (3). Recent studies suggest that a comprehensive evaluation should integrate anthropometric parameters (weight, height, BMI), body composition indices (Lean Tissue Index LTI, Fat Tissue Index FTI), serum biochemical markers (e.g., albumin, pre-albumin), the Dietary Inflammatory Index (DII), and clinical examinations.

Incorporating ASM as part of body composition and muscle mass assessment is valuable, particularly for diagnosing sarcopenia. The DII is a comprehensive dietary assessment tool that quantifies the overall inflammatory potential of an individual's diet by scoring various pro- and anti-inflammatory food components. Adjusting dietary patterns to lower the DII score may serve as an effective intervention strategy to delay CKD progression. When managing nutrition for CKD patients, controlling the dietary inflammatory potential is as crucial as ensuring adequate nutrient intake.

## 2 Stage-specific precision nutrition intervention: from early prevention to dialysis support

Early-stage CKD: Focus on an anti-inflammatory diet as the core strategy, controlling the DII score to delay the progression of a micro-inflammatory state. Plant dominant low-protein diets reduce the risk of developing incident CKD, CKD progression, and its related complications including cardiometabolic disease, metabolic acidosis, mineral and bone disorders, and uremic toxin generation (4). Simultaneously ensure adequate energy intake (30–35 kcal/kg/day) to delay disease progression and prevent malnutrition, while restricting sodium (< 4 g/day) and phosphorus (< 800 mg/day), and increasing dietary fiber to regulate gut microbiota (5).

Advanced CKD and Dialysis Patients: Attention should shift to specific nutrient supplementation to inhibit the overactivation of inflammatory pathways. Li, Zhao et al. found that supplementing with Bailing capsules combined with low-calcium dialysate in peritoneal dialysis patients significantly slowed the decline of residual renal function and improved micro-inflammation and oxidative stress. Lee et al. further revealed that both baseline and longitudinal Retinol-Binding Protein 4 (RBP4) levels in maintenance hemodialysis patients were negatively correlated with all-cause mortality, with the low RBP4 group having a 2.44-fold increased risk of death.

## 3 Sarcopenia prevention and treatment as a core objective in CKD management

Muscle atrophy affects over 50% of CKD patients and is significantly associated with morbidity and mortality. ASM (Romejko et al.) strongly correlates with anthropometric parameters (weight, BMI) and body composition (LTI, FTI), but not with renal function or traditional nutritional markers. This indicates that muscle loss occurs independently of conventional malnutrition indicators. Therefore, preventing and treating sarcopenia requires comprehensive strategies, including adequate high-quality protein intake, resistance exercise rehabilitation, vitamin D supplementation, and avoiding excessive protein restriction that could exacerbate muscle wasting.

## 4 Multidisciplinary collaboration: the essential path to achieving precision nutrition management

CKD nutritional management necessitates the integration of dietitians, rehabilitation therapists, and patient education. Future directions should include: Early Screening (Incorporate DII and body composition analysis into routine CKD assessments), Dynamic Monitoring (Adjust nutritional plans based on levels of RBP4 and inflammatory markers), and Whole-Process Management (Continuously optimize dietary structure and muscle mass maintenance from early CKD through the dialysis stage, dynamically adjusting strategies).

## 5 Conclusion

The nutritional management of chronic kidney disease has progressed beyond traditional nutrient restrictions into a multi-dimensional, individualized precision era. By integrating inflammation regulation (e.g., DII), dynamic biomarker monitoring (e.g., RBP4), and body composition assessment, a closed-loop management pathway of “monitoring-intervention-reassessment” can be constructed. This promotes a shift from a “one-size-fits-all” approach to “dynamic precision.” Future multi-center studies are needed to validate the clinical benefits of this pathway, ultimately enhancing patient quality of life and long-term prognosis.
